# IMProving care After inherited Cancer Testing (IMPACT) study: protocol of a randomized trial evaluating the efficacy of two interventions designed to improve cancer risk management and family communication of genetic test results

**DOI:** 10.1186/s12885-021-08822-4

**Published:** 2021-10-13

**Authors:** Deborah Cragun, Jason Beckstead, Meagan Farmer, Gillian Hooker, Marleah Dean, Ellen Matloff, Sonya Reid, Ann Tezak, Anne Weidner, Jennifer G. Whisenant, Tuya Pal

**Affiliations:** 1 College of Public Health, University of South Florida, 13201 Bruce B Downs Boulevard, IDRB 304, Tampa, FL 33612 USA; 2 My Gene Counsel, PO Box 612, Branford, CT 06405 USA; 3 Vanderbilt University Medical Center, 1500 21st Avenue South, Suite 2810, Nashville, TN 37212 USA; 4 Department of Communication, University of South Florida, 4202 East Fowler Avenue, CIS 3043, Tampa, FL 33620 USA; 5 Moffitt Cancer Center, 12902 USF Magnolia Drive, Tampa, FL 33612 USA

**Keywords:** Hereditary cancer, Genetic testing, BRCA, Lynch syndrome, Personalized medicine, Cancer risks, Management, Guideline adherence, Family communication, Clinical trial

## Abstract

**Background:**

Implementing genetic testing for inherited cancer predisposition into routine clinical care offers a tremendous opportunity for cancer prevention and early detection. However, genetic testing itself does not improve outcomes; rather, outcomes depend on implemented follow-up care. The IMPACT study is a hybrid type I randomized effectiveness-implementation trial to simultaneously evaluate the effectiveness of two interventions for individuals with inherited cancer predisposition focused on: 1) increasing family communication (FC) of genetic test results; and 2) improving engagement with guideline-based cancer risk management (CRM).

**Methods:**

This prospective study will recruit a racially, geographically, and socioeconomically diverse population of individuals with a documented pathogenic/likely pathogenic (P/LP) variant in an inherited cancer gene. Eligible participants will be asked to complete an initial trial survey and randomly assigned to one of three arms: A) GeneSHARE, a website designed to increase FC of genetic test results; B) My Gene Counsel’s Living Lab Report, a digital tool designed to improve understanding of genetic test results and next steps, including CRM guidelines; or C) a control arm in which participants continue receiving standard care. Follow-up surveys will be conducted at 1, 3, and 12 months following randomization. These surveys include single-item measures, scales, and indices related to: 1) FC and CRM behaviors and behavioral factors following the COM-B theoretical framework (i.e., capability, opportunity, and motivation); 2) implementation outcomes (i.e., acceptability, appropriateness, exposure, and reach); and 3) other contextual factors (i.e., sociodemographic and clinical factors, and uncertainty, distress, and positive aspects of genetic test results). The primary outcomes are an increase in FC of genetic test results (Arm A) and improved engagement with guideline-based CRM without overtreatment or undertreatment (Arm B) by the 12-month follow-up survey.

**Discussion:**

Our interventions are designed to shift the paradigm by which individuals with P/LP variants in inherited cancer genes are provided with information to enhance FC of genetic test results and engagement with guideline-based CRM. The information gathered through evaluating the effectiveness and implementation of these real-world approaches is needed to modify and scale up adaptive, stepped interventions that have the potential to maximize FC and CRM.

**Trial registration:**

This study is registered at Clinicaltrials.gov (NCT04763915, date registered: February 21, 2021).

**Protocol version:**

September 17th, 2021 Amendment Number 04.

## Background

Approximately 5–10% of cancers result from an inherited pathogenic variant in a cancer risk gene [1–6]. Implementing genetic testing for inherited cancer predisposition into routine clinical care offers a tremendous opportunity for cancer risk reduction and early detection for patients and their at-risk family members [7–14]. However, expanding testing options and the use of multi-gene panels may lead to more complex follow-up care [15]. Consequently, providers and patients may face challenges in implementing and engaging with guideline recommended care and avoiding overtreatment, particularly in the context of changing guidelines as new data become available [15]. Additionally, family communication (FC) of pathogenic/likely pathogenic (P/LP) variants with subsequent testing in family members can magnify the impact of genetic testing and prevent cancers among those at highest risk [16–19], yet multiple studies have shown FC and subsequent family testing remains limited [20–27].

Cancer risk management (CRM) guidelines are based on lifetime cancer risks, established cancer types associated with the underlying genetic cause, and the availability of effective cancer screening or risk reduction strategies. Using inherited breast cancer as an example, follow-up strategies to manage elevated breast cancer risks may include either enhanced breast cancer screening or bilateral mastectomy for high penetrance genes, such as *BRCA1/2* and *PALB2* [28]. In contrast, only heightened screening is recommended for those with P/LP variants in moderate penetrance genes, such as *ATM* and *CHEK2*; yet, prior research by us and others suggests that some women have bilateral mastectomies even though it may not be medically justified [29, 30]. Furthermore, while risk-reducing oophorectomy is recommended for *BRCA1/2* carriers around the age of 35–45, there is insufficient evidence to recommend this procedure for all women with *ATM*, *CHEK2*, or *PALB2* P/LP variants [28]; however, our prior study suggested that some of these women still have oophorectomies [29]. In addition to these examples of overtreatment, undertreatment has also been documented. For instance, annual colonoscopies are recommended among Lynch syndrome patients [31], yet in a prior study among Lynch syndrome patients, 32% of colonoscopies occurred > 27 months after their prior screen or > 3 months after receiving a positive genetic test result [32]. Another study demonstrated that only a quarter of individuals at high risk for Lynch syndrome realized they should have a colonoscopy every 1–2 years [33]. Additionally, engagement with guideline-directed CRM may vary by race/ethnicity [34] and decline over time [35]. Collectively, these findings highlight the need to test interventions to enhance engagement with guideline-based CRM and avoid both overtreatment and undertreatment.

Risk communication, cascade testing of family members, and risk-appropriate uptake of CRM options has the potential to vastly reduce cancer-related morbidity and mortality [16, 36]; however, data primarily from academic centers suggest that 20–40% of family members are not informed about positive genetic test results [23, 26, 27, 37]. Additionally, rates of family testing are substantially lower compared to overall rates of results disclosure [16, 17, 23, 27, 38–43], with two recent studies reporting that only 30% of family members had undergone testing [39, 42]. This may be due in part by the complexity of FC of genetic test results, which is often influenced by recognition of family members that are ‘at risk’, perceived responsibility to share, relationship type, physical or emotional closeness, perceptions of whether family members want to know, anticipation of family members’ reactions, personal emotions, and perceptions that discussing cancer is not accepted within the family [21, 25, 44, 45]. Additionally, given that over half of tested individuals are uncertain about the exact message to convey to their family members [46], it is critical to identify effective strategies to assist patients with FC [16, 18]. Furthermore, family members with whom P/LP variant results were shared underestimated their potential cancer risk and intentions to pursue genetic testing were low [47], highlighting the need to ensure messages used during FC of genetic test results convey the health threat and efficacy of actions that can reduce that threat. For example, we recently reported that in a sample of women with inherited breast cancer, most (94%) indicated they would find one or more resources helpful with FC and 70% reported using FC resources when telling family members about their genetic test result, including both printed and web-based information [43].

Given the gaps in implementation of guideline-directed CRM and potential threat of overtreatment and undertreatment, along with FC that has failed to result in high rates of genetic testing among at-risk family members, we propose a randomized trial to evaluate the effectiveness of two interventions — one focused on increasing FC and another focused on improving CRM. These interventions will be delivered to individuals with P/LP variants in inherited cancer genes across a racially, geographically, and socioeconomically diverse population. Data on implementation factors will be collected throughout the study to help ensure future delivery of interventions will maximize their impact by assuring or improving acceptability, appropriateness, and reach.

## Methods/design

### Trial design

**Fig. 1:**
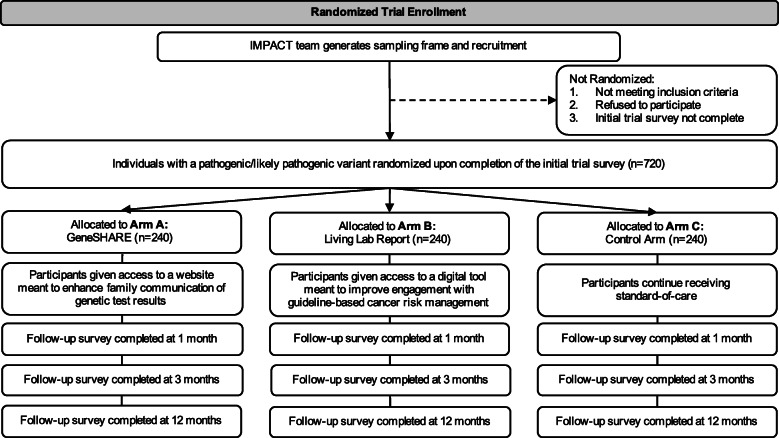
CONSORT flow diagram for the IMPACT study. CONSORT flow diagram showing participant flow through each stage of the hybrid type I randomized effectiveness-implementation trial (enrollment, intervention allocation, and follow-up)

Through recruitment of a racially, geographically, and socioeconomically diverse sample of individuals, this prospective, hybrid type I randomized effectiveness-implementation trial [48] (Fig. 1) will evaluate the effectiveness of two interventions that seek to improve FC of P/LP variant results and engagement with guideline-based CRM. After providing written informed consent, all trial participants will complete an initial trial survey and be randomly assigned to one of three arms: A) GeneSHARE, a website designed to increase FC; B) My Gene Counsel’s Living Lab Report, a digital tool designed to improve CRM; or C) a control arm in which participants will continue receiving standard care. Follow-up surveys will be conducted at 1, 3, and 12 months following randomization.

### Trial eligibility and recruitment

Approval to conduct human subjects research will be obtained from the Institutional Review Boards at Vanderbilt University (IRB # 202215) and the Tennessee Department of Health (IRB # 2021–0281). Eligibility criteria for the trial (Table 1) include individuals with a P/LP variant in an inherited cancer gene included in the Genetic/Familial National Comprehensive Cancer Network (NCCN) guidelines [28, 31]. Trial eligibility will be confirmed through self-reported baseline survey data and verification of genetic test results using a signed authorization for release of medical records. In recognition of their time, individuals who complete the baseline survey to determine trial eligibility will be offered a $10 gift card.

**Table 1:** Trial Eligibility

- English-speaking men and women aged 18 years or older	
- Not adopted (i.e., have information about their biological family members)	
- Have an email address with access to the internet and computer, tablet, or smartphone	
- Documented pathogenic/likely pathogenic (P/LP) variant in an inherited cancer gene with cancer risk management (CRM) guidelines listed in the National Comprehensive Cancer Network (NCCN) Genetic/Familial Guidelines on Breast, Ovarian, and Pancreatic or Colorectal cancers [28, 31]	
- Are non-adherent (i.e., either overtreatment or undertreatment) to at least one of the current NCCN CRM guidelines or if currently adherent, require ongoing cancer screening	
- Have at least one at-risk adult, living family member who either:	
° has not been told about the P/LP variant result by the participant	
° has not had their own genetic testing	

Individuals will be approached to complete the baseline survey to determine if they meet trial eligibility through two strategies: the Tennessee State Cancer Registry (TCR) and existing Vanderbilt studies. Using the two recruitment strategies we will enroll a total of 720 participants into the trial, with 240 participants randomized into each of the three arms.

Through the first recruitment strategy, eligible participants will be identified through the TCR, which is a centralized cancer registry that was established by law (Tennessee Code Annotated § 68-1-1001 [49]) and requires all healthcare providers and facilities that diagnose and/or treat cancer patients in Tennessee to report cancer case information to the Tennessee Department of Health [50]. Breast, colorectal, endometrial, and prostate cancer survivors aged ≥18 who meet NCCN criteria for inherited cancer genetic testing [28, 31] will be identified through the TCR and sent a letter by the TCR giving them an opportunity to opt out of participation. Three to 4 weeks after the letters are sent, contact information and relevant patient registry data (e.g., cancer diagnosis, treatment, screening, etc.) will be provided to the Vanderbilt research team on all potentially eligible individuals who do not opt out of being contacted. The research team will then proceed by mailing recruitment packets in batches to all rural dwellers and individuals who self-report as Black, as well as to randomly selected White/urban dwellers until yearly recruitment goals are met for racial and geographic strata. Recruitment packets will contain a contact letter, consent form, authorization for release of medical records, baseline survey, and a pre-paid, pre-addressed envelope to return the completed materials to the Vanderbilt research team. Based on our prior experience [34, 51], we expect that at least half of survey respondents will have had genetic testing, of which 5–10% will have a P/LP variant and be eligible to enroll in our trial [34]. We expect to contact ~ 4000 individuals to enroll approximately 240 participants through this strategy. This type of sampling approach has been used to effectively recruit racially, geographically, and socioeconomically diverse populations to our other studies [34, 51, 52].

Through the second recruitment strategy, we will approach individuals who participated in prior or ongoing Vanderbilt studies [34, 52–54], have confirmed P/LP variant results, agreed to be contacted for future research opportunities, have an email address, and are potentially eligible for the trial based on data obtained as part of the parent study. These individuals will be contacted via an email invite, which will include a link to the online consent form, authorization for release of medical records, and baseline survey. Through our existing and prior Vanderbilt studies, we currently have over 1500 participants who have a documented P/LP variant in an inherited cancer gene with NCCN guidelines [28, 31], confirmed through retrieval of genetic test results using a signed authorization for release of medical records as part of the parent studies. We estimate that more than half of these individuals will be eligible for the trial and with the ongoing recruitment to our existing research registry [53], we expect to reach our recruitment goal of approximately 500 trial participants through this recruitment strategy.

If an individual from either recruitment strategy does not respond within 2 weeks to the above attempts to contact them, a member of the Vanderbilt research team may send them reminder emails, mail additional recruitment packets, or contact the individual by phone to determine their interest in completing the baseline survey.

All potential trial participants, regardless of recruitment strategy, will be asked to review the baseline consent form and by completing the baseline survey each individual freely gives consent to participate.

### Trial procedures

Of the individuals who complete the baseline survey and are deemed eligible for the trial, we will purposefully invite 720 to participate in the trial, enriching for Blacks and rural dwellers, striving to ensure diversity based on gender, age, genetic test result, and cancer history. Select individuals will be invited to participate in the trial by an email invite, which will include a link to complete the online trial consent form developed in REDCap®, a secure, web-based, HIPAA-compliant, data collection platform [55]. Upon completing the online trial consent form, participants will be given the opportunity to download a PDF copy of the consent form for their records.

Once consented to the trial, participants will be asked to complete the online initial trial survey, which is also developed and housed in REDCap®. After survey completion, consented participants will be stratified according to gender (i.e., male versus female), personal history of cancer (i.e., yes versus no), race/ethnicity (i.e., non-Hispanic White versus other), and gene group (i.e., high penetrance cancer risk genes versus those that confer moderate cancer risks) and randomized (1:1:1) to one of the three arms of the trial (Fig. 1), with 240 participants per arm. Randomization will be performed by research staff using the randomization module available through REDCap® until recruitment goals are met. The third arm (Arm C) will serve as the control arm to the other two arms (Arms A and B) for their respective outcomes, which will be measured through clinical data review and online follow-up surveys completed at 1, 3, and 12 months following randomization. All trial participants will be offered a $20 gift card for completion of the online initial trial survey and a $20 gift card for completion of each of the three follow-up surveys (up to $80 for completing all four trial surveys).

### Trial interventions and primary outcomes

Participants assigned to Arm A will be provided a unique username/password to access the GeneSHARE website, developed based on our preliminary work related to FC among those with genetic testing for inherited cancer [29, 43, 45]. This website contains interactive and narrative components to enhance FC of genetic test results including: 1) an introductory video explaining genetic test results and the importance of testing among family members; 2) a family sharing letter and single-page handouts they can download, print, or email to assist in sharing genetic test results with at-risk family members; 3) a worksheet of reasons why others have shared test results instructing them to select or write in their own reasons for sharing; 4) a planning guide for listing when and how they will share results and follow-up with family members; 5) samples of how to start a conversation with family members and what to say if family members respond in a positive, unengaged, or negative fashion; 6) experiences of others who have overcome barriers to communicating with family members; and 7) supportive resources to help them address personal needs before and after sharing results. The primary outcome for the GeneSHARE intervention (Arm A) by the 12-month follow-up survey is either: 1) having at least one additional at-risk adult, living family member who has been told about the test result by the participant; or 2) among family members already aware of the test result, having at least one additional at-risk adult, living family member who the participant has subsequently followed up with about their test result, information about testing, or family history of cancer.

Participants assigned to Arm B will be provided a unique username/password to access Living Lab Report, a digital tool for patients developed by My Gene Counsel [56], which contains multiple resources including a summary of the patient’s genetic test results, condition-specific information, psychosocial support and resources, recommended CRM per NCCN guidelines, and information on accessing CRM services. The participant will also receive updates pertaining to their test result, including but not limited to changes in test result interpretation and updates to CRM guidelines or cancer risk information. Living Lab Report requires the uploading of genetic test results, with full functionality only available to those who are tested in a CLIA-approved lab. The primary outcome for the Living Lab Report intervention (Arm B) by the 12-month follow-up survey is ongoing or improved engagement with guideline-based CRM, the latter of which will be based on an increase in uptake of guideline-based CRM or a decrease in overtreatment. Engagement with guideline-based CRM is defined as individuals who are following NCCN guidelines based on genetic test results with no guideline-discordant care; all others will be classified as guideline-discordant.

Resources available through both interventions are designed to influence behavior change mechanisms and are aligned with the COM-B framework [57] and Theoretical Domains Framework (TDF) [58]. The COM-B framework, which is expanded by TDF to further elucidate constructs, was named after the following three essential conditions for behavior change: 1) Capability (knowledge and perceptions of ability); 2) Opportunity (social influences, environmental context, and available resources); and 3) Motivation (beliefs about consequences, social role, goals, emotions, and intentions) [57, 58]. Many TDF constructs to be measured throughout the trial are known mechanisms of action in other behavioral contexts such as handwashing, tobacco cessation, and mental health guidelines [58], providing an opportunity to measure facilitators and barriers to action for our participants.

Given the intervention includes no medical treatment, risk to participants is minimal. Any serious or unexpected adverse events will be submitted to the IRB within 7 days of the Principal Investigator receiving notification of the event. Research staff will be responsible for monitoring and reporting all adverse events that occur throughout the study. Additionally, the Vanderbilt-Ingram Cancer Center Data and Safety Monitoring Committee (VICC DSMC) will ensure the research achieves the highest quality standards. The VICC DSMC meets on a quarterly basis and ad hoc to discuss data and safety monitoring of clinical trials. Internal audits for compliance with adverse event reporting, regulatory and study requirements, and data accuracy and completion will be conducted according to the VICC Data and Safety Monitoring Plan according to the study phase and risk.

### Measures

Participants in all three trial arms will complete the same survey measures, which will include single-item measures, scales, and indices that have been adapted from several other studies (Table 2).

**Table 2:** Description of Study Measures

Behaviors and Behavioral Factors Associated with Family Communication (FC) and Cancer Risk Management (CRM)	
Behaviors (primary outcomes)	FC: Self-report of whether they shared their genetic test result with at least one family member for the first time or followed up with family (e.g., gave them relevant information, answered questions, etc.) [59]
	CRM: Self-report risk management behaviors verified via medical records review to determine if they are congruent with current NCCN guidelines [34, 60, 61]
Awareness and engagement in behavior	FC: Several items asking whether they have thought about sharing, chosen to share, and attempted to share and follow-up with family members (response options yes, no, unsure) [62]
	CRM: Single question asking them to pick one of seven statements that best describes where they are in following medical guidelines [63]
Capability (objective knowledge)	Both: Inherited cancer testing and treatment knowledge (scores range from 0 to 12 correct) [64, 65]
Capability (understand how to take action, plan & remember)	FC: Likert scale questions (know actions to take, know family members at high risk, clear plan of how, find behavior easy, can remember to act) [62, 66–72]
	CRM: Likert scale questions (know actions to take, clear plan of how, find behavior easy, remember to act) [62, 66, 68, 72]
Opportunity (social influences and environmental constraints/facilitators)	FC: Items asking if they think each relative would want to know the result (yes, no, unsure); Likert scale questions-normative influences of healthcare providers and family members; available resources (material resources, contact information, nothing preventing them from communicating) [62, 66–75]
	CRM: Likert scale questions-normative influences of healthcare providers, family/friends; available resources (insurance/money to cover cost, hospital or medical center, nothing preventing) [62, 68, 69, 72, 76–79]
Motivation (feelings and beliefs about the behavior)	FC: Likert scale questions (feelings about FC, important/useful, sense of duty to share information, anticipated outcomes of FC) [62, 66–73, 80, 81]
	CRM: Likert scale questions (feelings about guidelines, important/useful, anticipated outcomes of following guidelines) [62, 66, 69, 71–73, 80, 82]
Implementation Outcomes	
Acceptability	Intervention is satisfactory and agreeable - Acceptability of Intervention Measure (AIM) – 4-item Likert scale [83]
Appropriateness	Perceived fit, relevance, usefulness - Intervention Appropriateness Measure - 4-item Likert scale [83, 84]. Additional questions assessing perceived usefulness of various resources included in the respective interventions.
Exposure	Electronic data will capture what resources are viewed or downloaded and time spent on the website. Surveys will ask which resources they used and shared with family and healthcare providers.
Reach	RE-AIM steps for reporting on Reach [85] will be used – including extent to which participants and those who access resources as part of the interventions are representative based on key sociodemographic and clinical variables.
Implementation processes and cost	A systematic, theory-based approach described by Bunger et al. [86] will be used for tracking implementation processes (e.g., answering questions about the interventions, sending out email updates/reminders, etc.) and time involved maintaining the interventions. Costs can then be calculated, including time and website maintenance.
Other contextual factors	
Sociodemographic and clinical factors	1) Age, education, household income, marital/partner status, rural-urban community area code, living biological family members, health insurance status and type; 2) Personal/family history of cancer, stage of diagnosis; 3) Health Literacy [87]; 4) Gene with the pathogenic/likely pathogenic variant (verified by genetic test report); 5) Recommendations of their healthcare provider regarding behaviors
Family communication	Likert questions from Communication subscale of the McMaster Family Assessment Device [88]
MICRA	Uncertainty, distress, and positive aspects of genetic test results measured by the Multi-Dimensional Assessment of Cancer Risk Assessment (MICRA), Multiple Likert type items [89]

### Statistical analysis

A priori power analysis was conducted to estimate the sample size needed for testing hypotheses. Assuming a 15% difference in proportions (Arm A vs. Arm C, and Arm B vs. Arm C), 200 participants per arm will provide 80% power for testing hypotheses at the alpha = .05 level. Specifically, this sample size will be adequate for comparing a 15% difference in the proportion of participants who communicate with at least one additional at-risk family member by the 12-month timepoint (FC vs. control) and for comparing a 15% difference in the proportion of participants who improve or maintain engagement with guideline-based CRM by the 12-month timepoint (CRM vs. control). Assuming 16% attrition, based on pilot data, enrolling 240 per arm (total = 720) should result in 200 participants (total = 600) by the 12-month timepoint.

Demographic and clinical characteristics of participants will be summarized using descriptive statistics. Research staff will review survey data as it is received and contact participants to complete any missing data when possible. No differences are anticipated in the distribution of demographic and clinical characteristics between the groups given participants will be stratified by gender, personal history of cancer, race/ethnicity, and level of cancer risk conferred by the gene (i.e., high risk versus moderate risk) and then randomly assigned to groups. Nevertheless, the statistical significance of any observed differences in those variables will be assessed using the Chi-square/Fisher’s exact test for categorical variables and the two-sample t-test/Wilcoxon rank-sum test for continuous variables. Tests will be two-sided and if *p*-values are declared significant (≤0.05), variables will be adjusted for in subsequent analyses.

Logistic regression models will be used to compare differences in the behavioral outcomes between the control arm (Arm C) and each intervention arm (Arm A and Arm B) at the 12-month timepoint. Covariates will be assessed and added to the models as needed. Additionally, we will use the paired McNemar test to evaluate whether changes in the behavioral outcomes from the initial trial survey to the 12-month follow-up survey were significant in each arm.

Implementation outcomes for each intervention arm will be summarized using descriptive statistics. Exploratory analyses may be conducted to determine whether participants’ perceptions of acceptability and appropriateness of the interventions vary based on demographic characteristics or contextual factors.

Lastly, survey data will be analyzed using coincidence analysis (CNA) and the CNA package for R, following best practices outlined in a recently published study [90]. CNA will be used to reveal patterns of behavioral and contextual factors that systematically differ across individuals who did and did not achieve the primary outcomes for Arms A and B, respectively. CNA will uncover causal chains if they are substantiated by the data, allowing us to identify whether those who were engaged and reviewed specific intervention resources have higher levels of motivation, capability, or opportunity and whether these three behavioral factors contributed to the respective desired outcomes. Consequently, use of CNA is expected to identify which combinations of intervention resources work best, for which participants and test the COM-B model by assessing the impact intervention components have on capability, opportunity, motivation, and the behavioral outcomes.

## Discussion

The IMPACT study seeks to simultaneously evaluate two scalable interventions designed to improve outcomes of each respective intervention (i.e., FC of P/LP variant results and engagement with guideline-based CRM without overtreatment or undertreatment). This study is unique in that it will use a single control arm to simultaneously compare the effectiveness of two different interventions designed to impact two different behaviors that are both critical to maximizing the potential benefits from genetic testing for inherited cancer predisposition. The interventions target key behavioral change mechanisms (factors) based on our prior studies [29, 43], which we anticipate will contribute to their efficacy.

The IMPACT study, to our knowledge, will be the first to use CNA to gain a better understanding of how capability, opportunity, motivation, and use of intervention components may contribute in complex ways to FC and CRM behaviors and whether the key components needed for behavior change vary across demographic or clinical variables. We expect there will be differences in effectiveness of interventions based on various contextual factors and levels of engagement in the intervention. By determining what works for specific groups of individuals, we can better target these strategies to those for whom they are likely to be effective at improving CRM and FC. Given both interventions are information technology (IT)-based and scalable, they have the potential to reach large numbers of individuals at a low cost. We feel this pragmatic and efficient approach could maximize effectiveness and reach. Should we find that certain individuals or populations are not engaged with the automated, IT-based interventions we will consider additional, alternative resources and other methods to engage them to achieve the desired outcomes.

Last, this study leverages existing novel infrastructure that allows for the recruitment of patients that otherwise may be underrepresented in this type of study (i.e., Blacks, rural dwellers, individuals with low socioeconomic status indicators, and males). Leveraging the TCR offers a population-health approach to recruit participants with P/LP variants in inherited cancer genes. In contrast to the limited minority representation when sampling is based on a private insurer or academic institution, state cancer registries are also an effective means to recruit underserved populations such as Blacks, rural dwellers, and socioeconomically disadvantaged individuals who are more likely to have limited access to health services and/or be uninsured. In addition, through the Inherited Cancer Registry (ICARE), an existing Vanderbilt research registry, we partner with healthcare providers across the United States through various education and engagement activities related to inherited cancer predisposition, thus expanding recruitment beyond Tennessee [53]. In conclusion, we feel this highly innovative and practice-changing study will shift the paradigm by which individuals with P/LP variants in inherited cancer genes are provided with information to enhance FC of genetic test results and engagement with guideline-based CRM.

## Data Availability

Not applicable.

## Abbreviations

CNA: Coincidence analysis
CRM: Cancer risk management
FC: Family communication
IT: Information technology
NCCN: National Comprehensive Cancer Network
P/LP: Pathogenic/likely pathogenic
TCR: Tennessee State Cancer Registry
TDF: Theoretical Domains Framework
VICC DSMC: Vanderbilt-Ingram Cancer Center Data and Safety Monitoring Committee
